# Long-term functional outcomes of augmented primary repair using the peroneus brevis tendon for traumatic Achilles tendon ruptures: a retrospective cohort study

**DOI:** 10.1186/s12891-026-09668-2

**Published:** 2026-03-02

**Authors:** Duong Binh Tran, Thi Cao, Tu Van Phan

**Affiliations:** 1 Department of Orthopedics, Cho Ray Hospital, Ho Chi Minh City, Vietnam; 2 Department of Orthopedics and Rehabilitation, University of Medicine and Pharmacy at Ho Chi Minh City, Ho Chi Minh City, Vietnam

**Keywords:** Achilles tendon rupture, Peroneus brevis tendon, Tendon augmentation, Primary repair, Functional outcomes

## Abstract

**Background:**

Achilles tendon rupture is increasingly common and may result in significant functional impairment. Intraoperative findings such as frayed tendon ends, poor tendon quality, and larger tendon gaps may compromise the reliability of primary end-to-end repair alone. The purpose of this study was to evaluate the mid- to long-term functional outcomes of primary repair with peroneus brevis augmentation in closed traumatic Achilles tendon ruptures with unfavorable intraoperative findings.

**Methods:**

This retrospective cohort study included patients with closed traumatic Achilles tendon ruptures treated with primary end-to-end repair combined with peroneus brevis tendon augmentation. Demographic data, intraoperative characteristics, surgical techniques, and complications were reviewed. Functional outcomes were assessed using the Achilles Tendon Total Rupture Score (ATRS) and the American Orthopaedic Foot and Ankle Society (AOFAS) Ankle–Hindfoot score at final follow-up.

**Results:**

Thirty-five patients (36 Achilles tendons) were included, with a mean follow-up of 55.94 ± 32.52 months. Most ruptures demonstrated frayed tendon ends and tendon gaps greater than 2 cm. At final follow-up, functional outcomes were good to excellent, with mean ATRS and AOFAS scores of 93.28 ± 6.69 and 93.28 ± 7.69, respectively. All patients regained satisfactory ankle range of motion and plantarflexion strength, and were able to perform a single-heel rise. No tendon re-ruptures or major complications were observed.

**Conclusion:**

Primary end-to-end repair augmented with the peroneus brevis tendon provides reliable mid- to long-term functional outcomes for closed traumatic Achilles tendon ruptures with unfavorable intraoperative characteristics. This technique appears to be a safe and effective option to enhance repair strength and minimize the risk of re-rupture in selected patients.

## Introduction

Achilles tendon rupture is a common injury that predominantly affects the working-age population and has shown a steadily increasing incidence over recent decades [1, 2]. This condition often leads to significant functional impairment and prolonged rehabilitation, highlighting the importance of selecting an appropriate treatment strategy.

Both operative and nonoperative treatments have been widely investigated for acute Achilles tendon rupture. Recent randomized controlled trials and meta-analyses have reported comparable long-term functional outcomes between surgical and nonoperative management, while noting differences in re-rupture rates, complication profiles, and time to functional recovery [3–5]. Surgical treatment is therefore commonly considered in younger, physically active patients or in cases where early restoration of tendon continuity and strength is desired.

A variety of surgical techniques have been described for Achilles tendon repair, including open and minimally invasive approaches, as well as different suture configurations. However, certain intraoperative findings—such as frayed tendon ends, compromised tendon quality, and increased gap distance—may limit the reliability of primary end-to-end repair alone and increase the risk of tendon elongation or failure. In these situations, tendon augmentation or reconstructive techniques may be considered [6].

Several reconstructive options have been reported, particularly for chronic or neglected Achilles tendon ruptures, including V-Y tendon plasty, flexor hallucis longus tendon transfer, allograft reconstruction, and peroneus brevis tendon transfer [6–8]. Tendon transfer techniques, particularly flexor hallucis longus, have been widely reported with satisfactory outcomes in both acute and chronic Achilles tendon ruptures [9–11]. Among these techniques, peroneus brevis (PB) tendon transfer has been shown to provide satisfactory functional outcomes with low complication rates, especially in chronic Achilles tendon ruptures, as demonstrated in both endoscopic and open approaches [12, 13].

Although PB transfer is mainly reported for chronic ruptures [12–14], it may also be useful in acute cases with poor tendon quality or residual defects after debridement. In these situations, PB augmentation adds autologous tissue, reduces suture-line tension, and increases construct strength. Biomechanical studies have shown that local tendon transfers, including PB and flexor hallucis longus (FHL), can restore substantial mechanical strength. Unlike FHL transfer, PB augmentation preserves hallux plantarflexion function, as FHL harvest may affect hallux function (e.g., reduced/absent interphalangeal joint flexion), although clinically meaningful deficits may be limited [11], whereas PB transfer has been reported to cause minimal functional deficit [13] and still provides reliable reinforcement of the repair [14]. However, evidence for selective PB augmentation in acute closed traumatic ruptures remains limited; therefore, we evaluated mid- to long-term clinical and functional outcomes of primary repair with PB augmentation in this setting.

## Methods

This retrospective cohort study was conducted at the Department of Orthopedics and Trauma Surgery, Cho Ray Hospital, Ho Chi Minh City. Patients who underwent surgical repair of Achilles tendon rupture with PB tendon augmentation between 2016 and 2024 were identified from hospital records, with data extraction and follow-up assessment performed between January and October 2025. Patients were included only if the minimum follow-up was at least 12 months. As this was a retrospective observational study, prospective registration in a clinical trials database was not performed.

Patient data were retrieved from the hospital electronic database and archived medical charts, including demographic, clinical, imaging, operative, and postoperative information. Eligible patients were contacted for final follow-up evaluation. Patients unreachable after three attempts were considered lost to follow-up.

In this study, only patients who met the following criteria were included:

- Age ≥ 18 years.
- Closed traumatic Achilles tendon rupture. For all cases, the time from injury to surgery and the rupture location relative to the calcaneal insertion were recorded. Ruptures were classified as acute (< 2 weeks), subacute (2–6 weeks), and chronic (> 6 weeks).
- Intraoperatively confirmed unfavorable tendon characteristics, defined as markedly frayed or sclerotic tendon ends after debridement, inability to achieve secure end-to-end approximation in plantarflexion, or a residual gap despite maximal plantarflexion.
- Treated with primary repair augmented using the peroneus brevis tendon.
- Adequate medical records and follow-up for functional assessment.

Patients with prior Achilles or peroneal tendon surgery, associated vascular or complex foot and ankle injuries, active infection, pre-existing deformity or neurological impairment, inability to comply with rehabilitation, or incomplete data were excluded.

Surgical treatment in the present study reflects routine clinical practice; however, the analysis was intentionally restricted to patients with complete closed traumatic Achilles tendon rupture who underwent operative repair with PB tendon augmentation. Other surgically treated Achilles tendon injuries were not included.

All procedures followed a standardized operative protocol. Patients were positioned prone, and a longitudinal posterolateral incision was made along the lateral border of the Achilles tendon to expose the rupture site and assess tendon quality. Degenerated or frayed tissue was debrided to obtain healthy tendon ends suitable for repair. Then, tendon gap after debridement was measured in centimeters with the ankle in maximal plantarflexion.

**Fig. 1:**
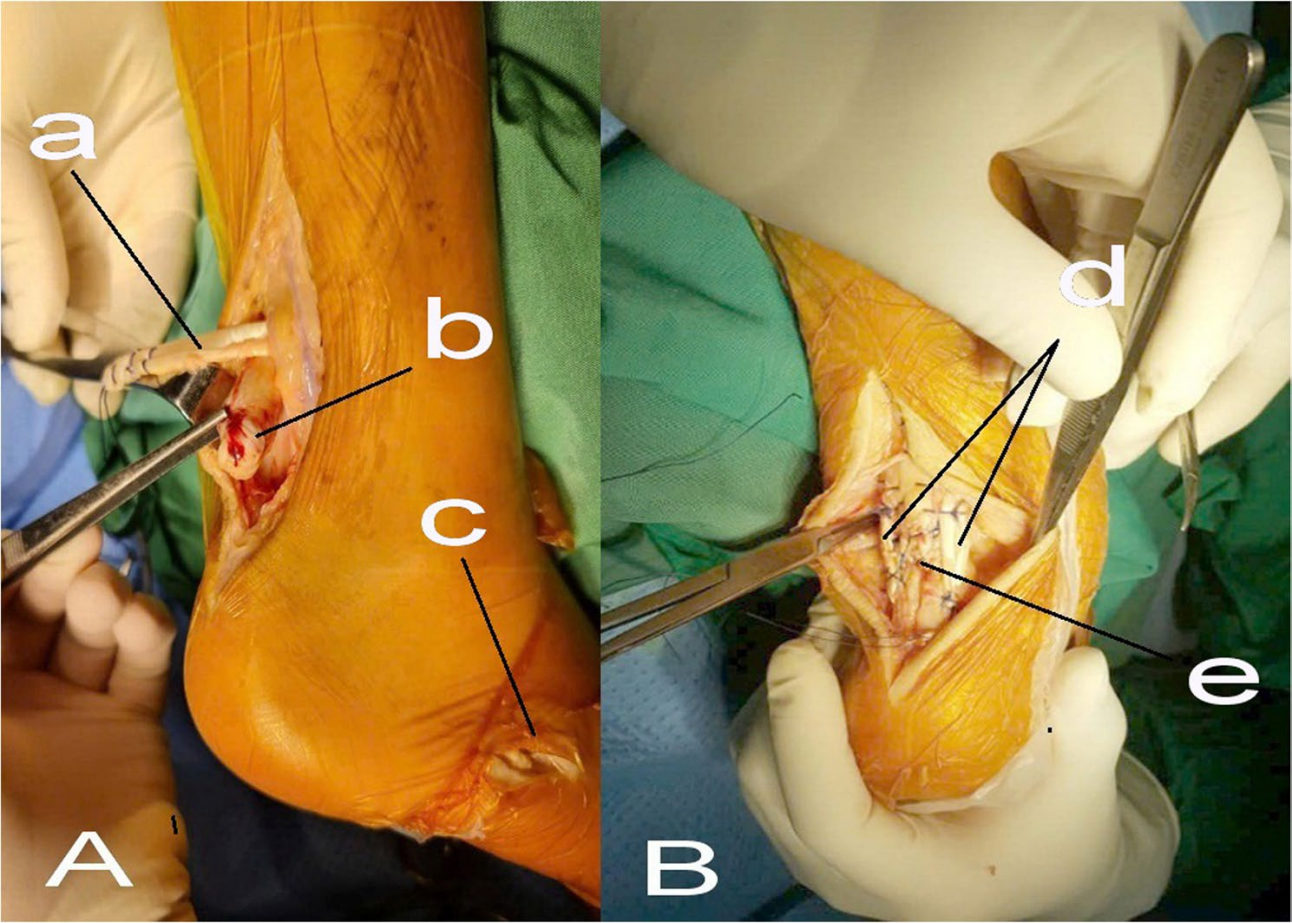
Harvest of the peroneus brevis tendon (**A**) and U-shaped passage of the peroneus brevis tendon through the Achilles tendon near its insertion (**B**). a: peroneus brevis tendon; b: ruptured Achilles tendon; c: peroneus brevis tendon harvest site; d: two limbs of the peroneus brevis tendon reinforcing both sides of the Achilles tendon repair; e: Achilles tendon suture site

Primary end-to-end repair of the Achilles tendon was performed using braided nonabsorbable sutures with various suture configurations, including the Krackow, Bunnell, and Kessler techniques. Augmentation using the peroneus brevis (PB) tendon was subsequently carried out to reinforce the primary repair. The PB tendon was harvested through a small distal incision at its insertion on the base of the fifth metatarsal, detached distally, and transferred proximally to the Achilles operative field (Fig. 1A). Transverse tunnels were created through the proximal and distal portions of the Achilles tendon, and the harvested PB tendon was passed through these tunnels either in a U-shaped configuration or, when sufficient length was available, in an O-shaped loop configuration to reinforce the repair zone (Fig. 1B). Fixation of the PB tendon to the Achilles tendon was completed using braided nonabsorbable sutures, while additional reinforcement sutures and soft-tissue closure were performed using absorbable braided sutures. In selected cases, when the distal Achilles tendon stump was too short to allow secure tendon-to-tendon fixation, generally less than approximately 3 cm, the harvested peroneus brevis tendon was anchored directly to the calcaneus using a suture anchor commonly used for rotator cuff repair. Intraoperative tension was adjusted to reproduce physiologic resting tension of the Achilles tendon, using the contralateral side as a reference. At the completion of the procedure, the wound was closed in layers, and the ankle was immobilized in a below-knee cast.

Postoperatively, the ankle was casted in plantarflexion for 2 weeks (non–weight-bearing). From weeks 2–6, plantarflexion was gradually reduced toward neutral (still non–weight-bearing). Weeks 6–8: neutral cast and progressive partial weight-bearing. Weeks 8–12: cast removed and progression to full weight-bearing as tolerated. After 12 weeks, supervised physiotherapy focused on range of motion, strength, endurance, and return to daily activities.

At final follow-up, functional recovery was evaluated using the Achilles Tendon Total Rupture Score (ATRS) [15] and the American Orthopaedic Foot and Ankle Society (AOFAS) Ankle–Hindfoot score [16]. The ATRS was self-administered by the patients, whereas the AOFAS score was completed by the treating surgeon based on clinical examination and patient responses. All assessments, including ankle range of motion (ROM) measured with a standard goniometer, were performed according to a standardized protocol at the final follow-up visit. Muscle strength was assessed using the Medical Research Council (MRC) grading system. Clinical examination assessed ankle range of motion in plantarflexion, dorsiflexion, inversion, and eversion, and the ability to perform a single-heel rise on the operated side. Postoperative complications, including wound-related problems, infection, tendon re-rupture, nerve injury, and thromboembolic events, were recorded.

## Results

### Patient characteristics

We reviewed 104 patients with Achilles tendon rupture. Of these, 55 patients were excluded because of open tendon injuries, non-traumatic ruptures, or alternative surgical techniques. The remaining 49 patients who underwent primary end-to-end repair combined with PB tendon augmentation met the inclusion criteria. Fourteen lacked follow-up data, resulting in 35 patients (36 closed traumatic Achilles tendon ruptures) included in the final analysis, with a mean follow-up of 55.94 ± 32.52 months. Demographic and injury characteristics are summarized in Table 1.

**Table 1:** Demographic and injury characteristics of the study population

Characteristic	Value
Number of patients	35
Number of Achilles tendons	36
Age at admission (years)	52.97 ± 12.73 (range: 20–75)
Sex	Male: 20 (57.1%); Female: 15 (42.9%)
Side of injury	Left: 17 (48.6%); Right: 18 (51.4%)
Number of injured tendons	Unilateral: 34 (97.1%); Bilateral: 1 (2.9%)
Mechanism of injury	Daily activities: 16 (45.7%); Sports-related: 15 (42.9%); Traffic accidents: 4 (11.4%)
Time from injury to surgery (days)	31.39 ± 37.97 (median 11; IQR 5–60)
Acute (< 2 weeks)	19 (52.8%)
Subacute (2–6 weeks)	7 (19.4%)
Chronic (> 6 weeks)	10 (27.8%)
Mean follow-up duration (months)	55.94 ± 32.52 (range: 12–108 months)

### Intraoperative characteristics of Achilles tendon injury

**Fig. 2:**
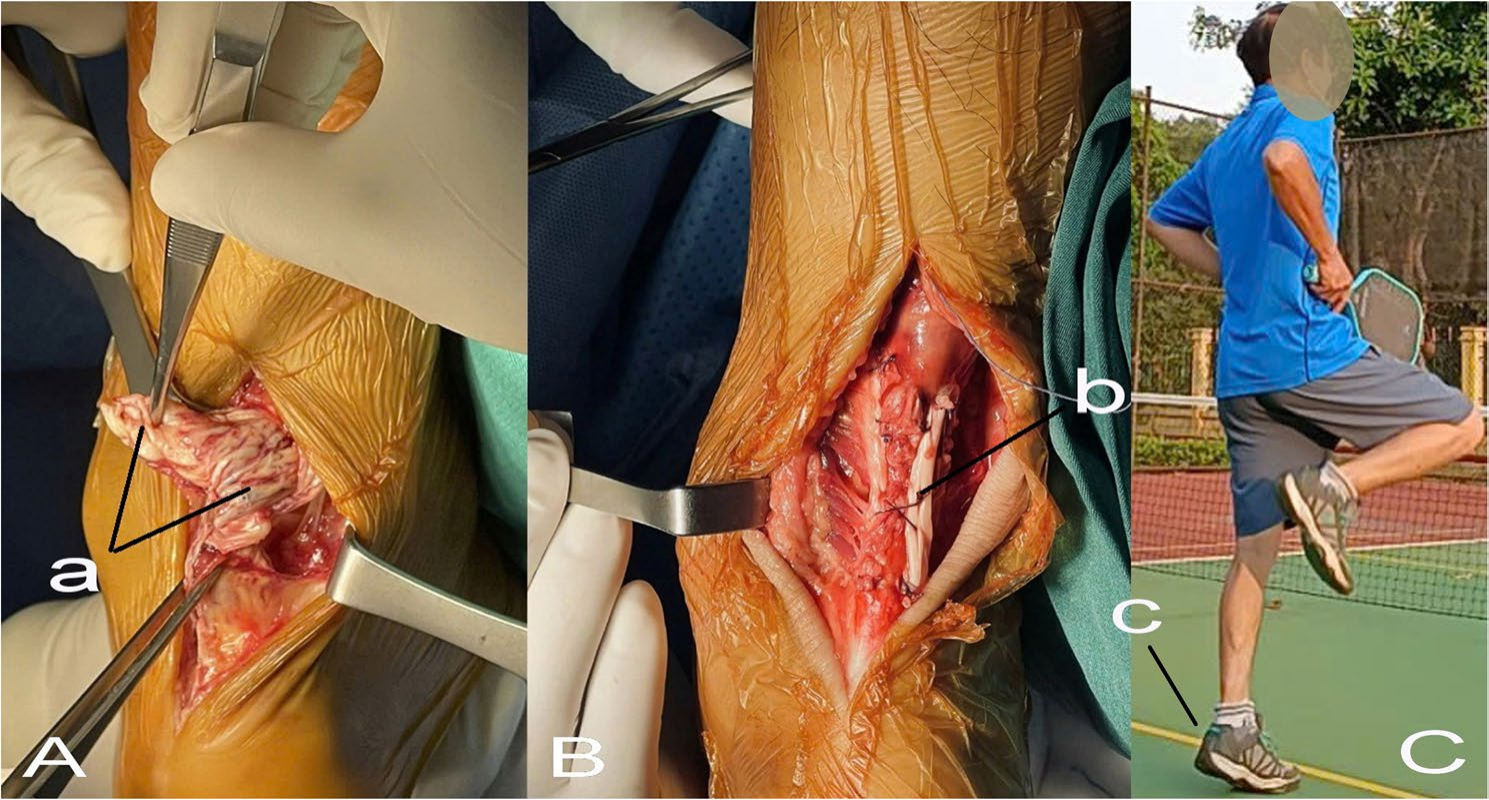
A representative case of a severely frayed Achilles tendon rupture with a large tendon gap (**A**), treated with peroneus brevis tendon augmentation (**B**), and the postoperative functional outcome (**C**). a: severely frayed Achilles tendon with a large defect; b: Achilles tendon repair reinforced with the peroneus brevis tendon; c: ability to perform a single-heel rise

Intraoperative findings demonstrated that most ruptures exhibited frayed tendon ends with a mean tendon gap of approximately 3 cm (Fig. 2A), and more than one-third of cases showed tendon sclerosis. The majority of ruptures were located within the watershed region (2–5 cm from the calcaneal insertion), supporting the indication for tendon augmentation. Detailed intraoperative characteristics are summarized in Table 2.

**Table 2:** Intraoperative characteristics of Achilles tendon injury

Characteristic	Total (*n* = 36)	Percentage (%)
Type of anesthesia		
General anesthesia	19	52.8
Spinal anesthesia	17	47.2
Gap between tendon ends after debridement with the ankle in plantarflexion (cm)	3.09 ± 1.23	—
≤ 2 cm	11	30.6
> 2 cm	25	69.4
Paratenon rupture	0	0
Rupture pattern (frayed tendon ends)	36	100
Tendon sclerosis		
Present	14	38.9
Absent	22	61.1
Distance from calcaneal insertion (cm)	4.04 ± 1.12	—
< 2 cm	2	5.6
2 to < 4 cm	12	33.3
4 to < 5 cm	15	41.7
5 to < 6 cm	4	11.1
≥ 6 cm	3	8.3

### Intraoperative surgical techniques

The surgical techniques applied during Achilles tendon reconstruction are summarized in Table 3. The mean length of the PB tendon harvested for augmentation was 13.64 ± 1.08 cm, ranging from 11.5 to 15 cm. Regarding the tendon repair configuration, the Krackow technique was the most frequently used method, accounting for 75.0% of cases, followed by the Bunnell technique in 22.2% and the Kessler technique in 2.8%. Fixation of the PB tendon to the calcaneus using a suture anchor was required in a minority of cases (8.3%). The mean operative time was 101.11 ± 24.53 min, with a range from 55 to 150 min.

**Table 3:** Surgical technique characteristics (*n* = 36 tendons)

Characteristic	Total (*n* = 36)	Percentage (%)
Length of peroneus brevis tendon used (cm)	13.64 ± 1.08	—
Tendon repair technique		
Krackow technique	27	75.0
Bunnell technique	8	22.2
Kessler technique	1	2.8
Calcaneal anchor fixation		
Yes	3	8.3
No	33	91.7
Operative time (minutes)	101.11 ± 24.53	—

### Functional outcomes

At final follow-up, ATRS and AOFAS scores indicated good-to-excellent functional recovery (Table 4). When stratified by chronicity, 19 tendons were classified as acute, 7 as subacute, and 10 as chronic. Mean ATRS scores were 95.37 in the acute group, 92.57 in the subacute group, and 89.80 in the chronic group. Corresponding mean AOFAS scores were 96.11, 93.29, and 87.90, respectively. Most patients demonstrated near-normal ankle range of motion and plantarflexion strength on clinical examination. All patients were able to perform a single-heel rise on the operated side and returned to daily activities without major limitations.

**Table 4:** Functional outcomes at final follow-up (*n* = 36 Achilles tendons). Values are presented as mean ± standard deviation. 95% confidence intervals are provided for the ATRS and AOFAS scores

Outcome	Value	*n* (%)	95% CI
Muscle strength (MRC grade 5/5)		36 (100)	
Ankle plantarflexion ROM (degrees)	50.92 ± 2.77		
Ankle dorsiflexion ROM (degrees)	18.19 ± 2.13		
Ankle inversion ROM (degrees)	28.64 ± 1.52		
Ankle eversion ROM (degrees)	14.03 ± 1.13		
AOFAS Ankle–Hindfoot score	93.28 ± 7.69		90.67–95.88
AOFAS category: Excellent (90–100)		23 (63.9)	
AOFAS category: Good (80–89)		13 (36.1)	
ATRS	93.28 ± 6.69		91.01–95.54
ATRS category: Excellent (90–100)		22 (61.1)	
ATRS category: Good (80–89)		14 (38.9)	

### Complications

Postoperative complications were infrequent. Superficial wound infections were observed in 11.1% of cases, and all were successfully managed with conservative treatment, without the need for reoperation. No tendon re-ruptures were observed (0/36; 95% CI, 0–9.74%). No cases of nerve injury, deep vein thrombosis, or other major complications were recorded during the follow-up period.

## Discussion

The present study evaluated the mid- to long-term outcomes of primary end-to-end repair combined with PB tendon augmentation in patients with closed traumatic Achilles tendon rupture, with a mean follow-up of 55.94 ± 32.52 months (range: 12–108 months). The interval from injury to surgery varied considerably, with the longest delay being 120 days after the initial injury. Previous studies have reported that delayed repair performed between 14 and 30 days after injury can yield outcomes comparable to those of acute repair [17].

Overall, the results of this series demonstrated consistently favorable outcomes. Functional recovery was good to excellent, as reflected by high ATRS and AOFAS scores, and no tendon re-ruptures or major complications were observed during follow-up. Intraoperative findings showed a predominance of frayed tendon ends, moderate tendon gaps, and rupture sites within the watershed zone, supporting the rationale for tendon augmentation in these cases.

### Functional outcomes and clinical relevance

In the present cohort, patients demonstrated excellent functional recovery at final follow-up, as reflected by consistently high ATRS and AOFAS scores. In previously published studies, mean ATRS values after standard surgical repair typically range from 79 to 83 at 12 months, as reported by Olsson et al. [18] and in the large randomized controlled trial by Myhrvold et al. [4]. In contrast, the markedly higher ATRS scores observed in the present study are comparable only to a limited number of reports, such as that of Zou Y. et al., who reported a mean ATRS of 98.3 ± 9.2 following double-bundle FHL transfer for chronic Achilles tendon ruptures [19].

In the present study, the mean AOFAS Ankle–Hindfoot score was 93.28 ± 7.69, with all patients achieving good or excellent outcomes, indicating favorable recovery of ankle–hindfoot function after Achilles tendon rupture repair. As a composite measure incorporating pain, functional performance, and alignment, the AOFAS score reflects both subjective and objective aspects of postoperative recovery. Wu et al. (2023) reported a mean AOFAS score of 93.6 ± 3.4 at 2-year follow-up, which is closely comparable to our findings [20]. In another study, Bashir et al. demonstrated substantial postoperative improvement, with mean AOFAS scores increasing from 77.9 ± 4.3 at 3 months to 96.16 ± 1.1 at 12 months following surgical repair of Achilles tendon rupture, with all patients achieving good or excellent outcomes at final follow-up [21]. Overall, the AOFAS outcomes observed in the present study are consistent with the favorable functional recovery reported in the literature. Large randomized trials and meta-analyses have shown that surgical repair provides lower re-rupture rates and faster functional recovery than nonoperative treatment, particularly in active patients [3, 4]. Despite these favorable outcomes, concerns remain regarding wound complications and long-term strength deficits after surgery. The consistently high ATRS and AOFAS scores observed in this study suggest that PB tendon augmentation may enhance functional recovery by reinforcing the primary repair and improving load sharing across the repair site (Table 4). Importantly, at final follow-up, all patients were able to perform a single-heel rise on the operated side, indicating satisfactory restoration of plantarflexion strength. The test was considered successful when the patient was able to perform at least one unsupported heel rise, with the heel clearly lifted off the ground.

### Rationale for peroneus brevis tendon augmentation

Intraoperative assessment revealed that all ruptures exhibited frayed tendon ends, with a mean tendon gap of 3.09 ± 1.23 cm, and 69.4% of cases presenting with gaps greater than 2 cm (Table 2). These characteristics are commonly encountered in closed traumatic Achilles tendon ruptures and may compromise the mechanical strength and reliability of primary end-to-end repair alone. Previous studies have emphasized that poor tendon quality, frayed rupture patterns, and larger tendon gaps are associated with an increased risk of elongation or failure following primary repair [7]. Jiménez-Carrasco et al. reported the use of allograft reconstruction for tendon defects larger than 7 cm and autologous grafts for defects exceeding 4 cm in neglected Achilles tendon ruptures, highlighting the importance of augmentation strategies when primary repair is insufficient to bridge the defect or ensure adequate stability [22]. In the present study, all ruptured tendons demonstrated frayed ends with varying degrees of severity, ranging from minor (Figs. 1) to more extensive fraying (Figs. 2), reflecting compromised tendon quality observed intraoperatively. These unfavorable characteristics further support the rationale for selective tendon augmentation in cases where primary repair alone may be biomechanically inadequate.

Peroneus brevis tendon augmentation has been advocated as a reliable solution in such situations. The classic work by Turco and Spinella described the use of PB tendon transfer for Achilles tendon rupture, a technique originally introduced by Perez Teuffer in 1971, with satisfactory functional outcomes reported [14]. More recent studies by Maffulli and colleagues have demonstrated durable long-term results following PB tendon transfer for chronic Achilles tendon tears, with no reported re-ruptures and acceptable functional performance at extended follow-up [13]. Similarly, minimally invasive and endoscopic PB transfer techniques have shown encouraging outcomes in chronic and neglected ruptures, with high patient satisfaction and low complication rates [12].

Although PB tendon augmentation has been more commonly reported in chronic or neglected Achilles tendon ruptures [12–14], the findings of the present study suggest that selective augmentation may also be beneficial in acute-to-subacute closed traumatic ruptures presenting with unfavorable intraoperative characteristics. This approach may provide additional mechanical stability while preserving native Achilles tendon continuity through primary end-to-end repair.

When stratified according to the time-based classification used in this study, functional outcomes showed a gradual decline with increasing chronicity, with the highest ATRS and AOFAS scores observed in the acute group, followed by the subacute and chronic groups. This trend suggests that earlier surgical intervention and more favorable tendon conditions might be associated with better functional recovery. However, it should be noted that the three-stage classification (acute, subacute, and chronic) used in this study was adopted for clinical analysis purposes. In the existing literature, most references do not formally distinguish three stages, and ruptures are generally considered chronic when diagnosis or treatment is delayed beyond approximately 4–6 weeks after injury [6].

### Surgical technique considerations

The predominance of the Krackow suture technique in this series reflects its well-documented biomechanical advantages, including superior resistance to gap formation and higher load to failure compared with other suture configurations. The mean operative time was acceptable and comparable with previously reported series of augmented Achilles tendon repair. Fixation of the PB tendon to the calcaneus using a suture anchor was required in only a small proportion of cases, indicating that augmentation could often be achieved without additional bony fixation.

Importantly, no major complications related to PB tendon harvest were observed. Preservation of ankle stability and eversion strength after PB transfer has been a concern; however, previous studies have shown minimal long-term functional deficits following PB tendon transfer, particularly when the peroneus longus tendon remains intact [13].

### Complications and safety profile

Postoperative complications were infrequent in the present series. Superficial wound infections occurred in 11.1% of cases and resolved with conservative management, without the need for reoperation. This rate is higher than those reported in large randomized trials and meta-analyses, where Ochen et al. reported an infection rate of 2.8% after surgical treatment [3], and Myhrvold et al. reported deep infection rates of 1.1% following open repair and 1.7% following minimally invasive techniques [4]. However, our findings are comparable to those of Berk Güçlü et al., who reported superficial wound infections in approximately 11% of patients, all resolving with oral antibiotics [23]. The infection profile observed in this study falls within the range reported for open Achilles tendon repair and was not associated with deep infection. No cases of re-rupture, nerve injury, or thromboembolic events were recorded. The absence of re-rupture in the present study is noteworthy and may reflect the combined benefits of reinforced repair, careful patient selection, and structured postoperative rehabilitation.

### Study limitations

Several limitations of this study should be acknowledged. First, because of the retrospective design and the single-time-point follow-up assessment, formal test–retest reliability analysis of the outcome measures was not performed. In addition, the absence of a control group treated with primary end-to-end repair alone limits direct comparison of treatment efficacy. The absence of a control group also limits the ability to attribute the favorable outcomes specifically to the augmentation procedure. Therefore, the results should be interpreted as descriptive outcomes of a selective augmentation strategy in patients with unfavorable intraoperative tendon characteristics, rather than as evidence of superiority over primary repair alone. Second, the relatively small sample size and the absence of an a priori power analysis limit the statistical power of the study, particularly for detecting rare complications such as re-rupture. Third, the single-center setting may limit the generalizability of the findings. Fourth, selection bias cannot be excluded, as only patients with unfavorable intraoperative characteristics who underwent augmentation and had sufficient follow-up were included, potentially leading to an overestimation of functional outcomes. Fifth, there was heterogeneity in the time from injury to surgical intervention, with patients presenting across an acute-to-subacute spectrum and a subset undergoing delayed surgery, which may have influenced healing potential and functional recovery. Sixth, objective assessments such as isokinetic strength testing or gait analysis were not performed, and subtle functional deficits may therefore have been underestimated. In addition, return-to-work or return-to-sport data were not systematically collected, and donor site morbidity after peroneus brevis harvest was not objectively assessed, which may limit the evaluation of real-world functional impact. Finally, the influence of the surgeon learning curve over the study period cannot be excluded, as technique refinement may have affected outcomes. Despite these limitations, the present study provides valuable clinical data on a selective PB tendon augmentation strategy in closed traumatic Achilles tendon ruptures, supported by detailed intraoperative findings and extended follow-up.

## Conclusion

Primary end-to-end repair combined with peroneus brevis tendon augmentation for closed traumatic Achilles tendon rupture resulted in good to excellent functional outcomes, a low complication rate, and no re-ruptures at mid- to long-term follow-up. Intraoperative findings of frayed tendon ends, moderate tendon gaps, and rupture location within the watershed zone support the rationale for selective augmentation. This technique appears to be a safe and feasible surgical option for Achilles tendon ruptures with unfavorable intraoperative characteristics and may contribute to improved functional recovery in selected patients.

## Data Availability

The datasets generated and analyzed during the current study are available from the corresponding author upon reasonable request.
